# SPARSE 1.0: a template for databases of species inventories, with an open example of Czech birds

**DOI:** 10.3897/BDJ.11.e108731

**Published:** 2023-11-23

**Authors:** Kateřina Tschernosterová, Eva Trávníčková, Florencia Grattarola, Clara Rosse, Petr Keil

**Affiliations:** 1 Czech University of Life Sciences Prague, Praha - Suchdol, Czech Republic Czech University of Life Sciences Prague Praha - Suchdol Czech Republic; 2 German Centre for Integrative Biodiversity Research (iDiv) Halle-Jena-Leipzig, Leipzig, Germany German Centre for Integrative Biodiversity Research (iDiv) Halle-Jena-Leipzig Leipzig Germany

**Keywords:** aves, biodiversity informatics, open data, nature reserve, re-survey, sample area, time series, checklist, survey

## Abstract

Here, we introduce SPARSE (acronym for "SPecies AcRoss ScalEs"), a simple and portable template for databases that can store data on species composition derived from ecological inventories, surveys and checklists, with emphasis on metadata describing sampling effort and methods. SPARSE can accommodate resurveys and time series and data from different spatial scales, as well as complex sampling designs. SPARSE focuses on inventories that report multiple species for a given site, together with sampling methods and effort, which can be used in statistical models of true probability of occurrence of species. SPARSE is spatially explicit and can accommodate nested spatial structures from multiple spatial scales, including sampling designs where multiple sites within a larger area have been surveyed and the larger area can again be nested in an even larger region. Each site in SPARSE is represented either by a point, line (for transects) or polygon, stored in an ESRI shapefile. SPARSE implements a new combination of our own field definitions with Darwin Core biodiversity data standard and its Humboldt core extension. The use of Humboldt core also makes SPARSE suitable for biodiversity data with temporal replication.

We provide an example use of the SPARSE framework by digitising data on birds from the Czech Republic, from 348 sites and 524 sampling events, with 15,969 unique species-per-event observations of presence, abundance or population density. To facilitate use without the need for a high-level database expertise, the Czech bird example is implemented as MS Access .accdb file, but can be ported to other database engines. The example of Czech birds complements other bird datasets from the Czech Republic, specifically the four gridded national atlases and the breeding bird survey which cover a similar temporal extent, but different locations and spatial scales.

## Description

SPARSE database is designed to store species inventory data, with special emphasis on documenting sampling effort and methods and can accommodate considerable variation in spatial configuration of inventories, as well as multiple repeated inventories done at the same site at different times (a.k.a. time series). The database has simple structure, so that others can use it or copy it, without a detailed knowledge of advanced database environments. This is also the reason for implementing it in MS Access, which is widespread and user-friendly, albeit commercial, software. Each site in the Access database is represented by a point, line or polygon in ESRI shapefiles that are provided separately from the Access file; these two data types (Access tables and ESRI shapefiles) are linked using the *objectID* identifier unique to each site.

When designing SPARSE, we used a combination of Darwin core (DWC, Wieczorek et al. (2012)) and Humboldt core (HC, Guralnick et al. (2017)) biodiversity data standards. This was to make the database as inter-operable with other datasets as possible. On top of DWC and HC, however, we also defined our own fields; this was sometimes necessary when no suitable equivalent was available in HC or DWC.

SPARSE is designed to be modular and customisable, but we also needed it to maintain integrity and quality of the data. For this purpose, the current version of SPARSE comes with a set of controlled vocabularies in 15 codebooks. However, their use in any future derivatives of SPARSE is completely optional and they can easily be removed or modified.

SPARSE structure should work for groups of organisms other than birds and should be applicable to different regions of the world, as well as for various sampling methods and measurements. The structure can be readily converted to other database engines, such as PostgreSQL.

## Introduction

Thanks to initiatives such as GBIF, eBird or iNaturalist, the volume of biodiversity data has been growing (https://www.gbif.org/analytics/global), particularly the volume of **presence-only incidental observations**. However, these data have important drawbacks: (1) Although GBIF and eBird have the option to record where a species was *not* recorded, this is still not a common practice. At the time of writing of this manuscript, absences made ca. 1% of GBIF records (there were 27 million absences vs. 2,582 million presences). This lack of absences limits the use of the data in probabilistic species distribution models (Pearce and Boyce 2006); (2) Presence-only data are usually not recorded as time series (i.e. as repeated surveys at the same location with known sampling effort), which limits their use in analyses of temporal biodiversity change; (3) they have pronounced geographic biases (Meyer et al. 2015). A data type that can potentially address these limitations are species inventories, surveys and faunistic or floristic checklists (hereafter **inventories**). An inventory is an event during which one or more experts survey a given site for all species, the experts reporting their survey methodology and effort, together with the list of species that they detect, sometimes with information on abundances or population densities. The site can either be a point (an expert stands at a spot and detects species, such as birds), a linear transect or a more complex shape represented by a polygon of a given area. Examples of inventories are surveys of natural reserves (e.g. Kadlec et al. 2008, Bürger and Kloubec 1991) or ecological surveys for species present at a location that is designated for a major construction activity.

Unlike presence-only point records, inventories can potentially be used in statistical models assessing probability of occurrence (Pearce and Boyce 2006) and, consequently, also to calculate variables such as species richness and other specific metrics of biodiversity (Keil and Chase 2019). Inventories also sometimes come with temporal dimension - for example, a survey of a protected area might have been repeated after some time (e.g. Bürger and Kloubec 1991, Kadlec et al. 2008), which makes inventories of interest for studies of biodiversity dynamics. A seeming drawback of inventories is that they vary massively in the area that they cover. However, this can also be an advantage; when an area of an inventory is known exactly (which it usually is), it can be used as a covariate in statistical models of scale-dependent phenomena such as biodiversity gradients (Keil and Chase 2019) or species-area relationships (Storch and Kotecky 1999). Finally, inventories can be found in areas not covered by other types of biodiversity data, particularly inventories of countries or counties; an example is a series of coarse-grain checklists of dragonflies in African countries (Sandall et al. 2022), for which fine-grain local data are unavailable.

The challenge is how to mobilise and store such heterogeneous data as inventories (König et al. 2019), since each inventory comes with a different area, geometry of the area sampled, sampling methods and efforts, temporal coverage and taxonomic scope. Inventories sometimes have complex nested designs where an inventory of a nature reserve (represented by a polygon) consists of a series of transects, which then consist of a series of point observations. To address this challenge, we introduce **SPARSE** (acronym for "SPecies AcRoss ScalEs"), a database framework that can store data on species composition derived from ecological inventories, surveys and checklists, with emphasis on metadata describing sampling effort and methods. SPARSE can accommodate re-surveys and time series and data from different spatial scales, as well as complex sampling designs. Although some of this functionality is present in GBIF, the original focus of GBIF was not data coming from inventories, nor complex spatial designs with temporal replication. This is also why the idea of Humboldt core has been proposed as an extension of the original data model of GBIF (Guralnick et al. 2017). However, Humboldt core has not yet been fully implemented, nor has it been a part of GBIF. Furthermore, on the conceptual level, the Humboldt core, as it is described in Guralnick et al. (2017), still lacks some of the functionality that we require in our research group, particularly the ability to handle complex spatial designs and spatial scale. That is why we have created SPARSE.

**Figure 1.**
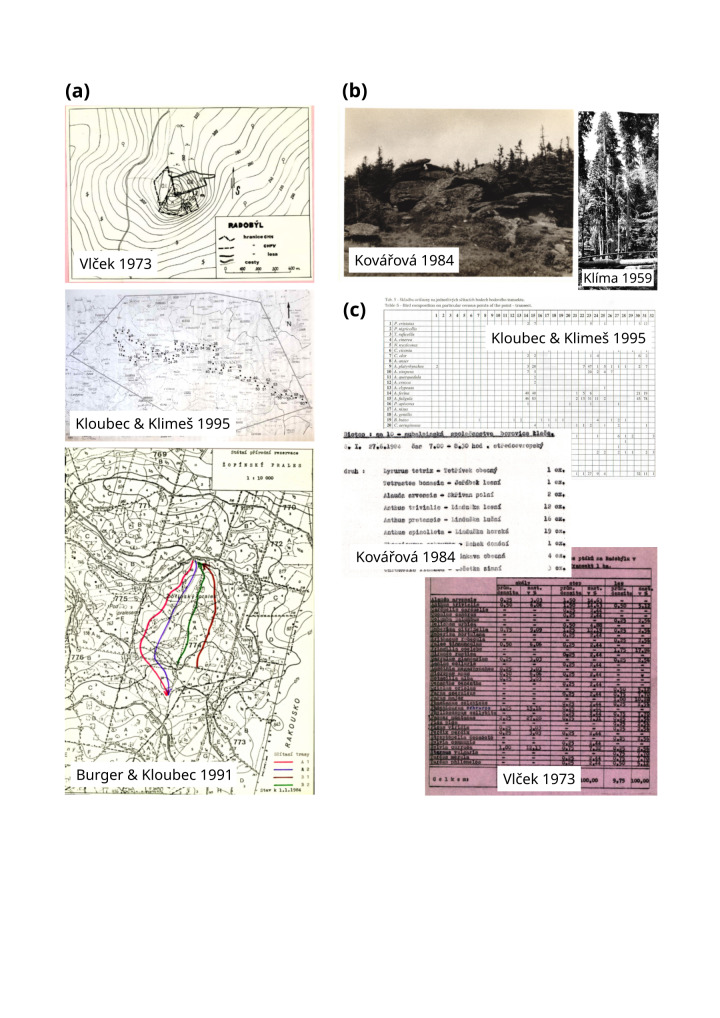
Examples of valuable raw contents of Czech bird inventories that were published during the 20^th^ century in local journals and white papers. **a** Some published inventories come with detailed maps of surveyed areas, sometimes with complex sampling designs, such as the points located along a transect within a polygon (Kloubec and Klimeš 1995); **b** Some publications contain photographs of the surveyed habitats, which can be used to extract information about percentage of land-cover types, such as forest, something that we have implemented here in SPARSE; **c** Examples of how species composition across sites is presented in different publications. This varies from barely legible and loosely structured typewriter texts to well organised spreadsheets.

To provide an example of how SPARSE can work, we used it to store species inventory data on Czech birds. These mostly consist of inventories of natural reserves and faunistic surveys of various patches of habitats (e.g. for a specific forest) published in local journals or as white papers (Fig. 1). Many of these inventories have been partially aggregated and digitised by the open Czech NDOP database (https://portal.nature.cz/nd/), but they lack some key metadata on sampling methods and effort. Besides NDOP, there have been hundreds, or even thousands, of inventories published during the second half of the 20^th^ century in grey literature, regional journals, white papers and government reports. We have digitised these and we supplemented them with inventories from NDOP. For the latter, we also extracted information on sampling methods and effort, which has not been done so far.

All of this is readily available and can be downloaded and edited by users as they please. Thus, SPARSE is not a centralised database where contributors upload their data. Instead, its purpose is to serve as a simple template that is stored on users' personal computers and which users can modify for their own projects, for example, by adding new fields or vocabularies.

By focusing on Czech birds, we hope to provide an additional source of data that complements other existing bird databases. Czech Republic has some of the best high-quality presence-absence and abundance bird data in the world. This includes four periods of gridded atlas data at resolutions of ca. 10 x 10 km^2^ (Hudec et al. 1987, Šťastný et al. 1997, Bejček et al. 2006, Šťastný et al. 2021) and several hundred local transects surveyed over several decades (http://jpsp.birds.cz/, Reif et al. (2013)). Example uses of these data, with more details, are in Koleček et al. (2010) or Reif et al. (2022). Unfortunately, these data are not open. Furthermore, they do not cover intermediate spatial scales from ca. 1 x 1 km^2^ up to the atlas data of 10 x 10 km^2^. Conveniently, the bird dataset that we present here provides data from exactly these spatial scales.

## Implementation

SPARSE consists of the main MS Access file (*SPARSE.accdb*), an MS Excel spreadsheet with detailed field definitions (*SPARSE_definitions.xlsx*), an .xlsx template for data input (*INPUT_data-template.xlsx*), a BibTeX file with complete bibliography of all studies in the database (*SPARSE_bibliography.bib*), a *SPARSE_shapefile* folder that lists all the points, lines and polygons corresponding to sites and a code for processing and plotting the data.

**Figure 2.**
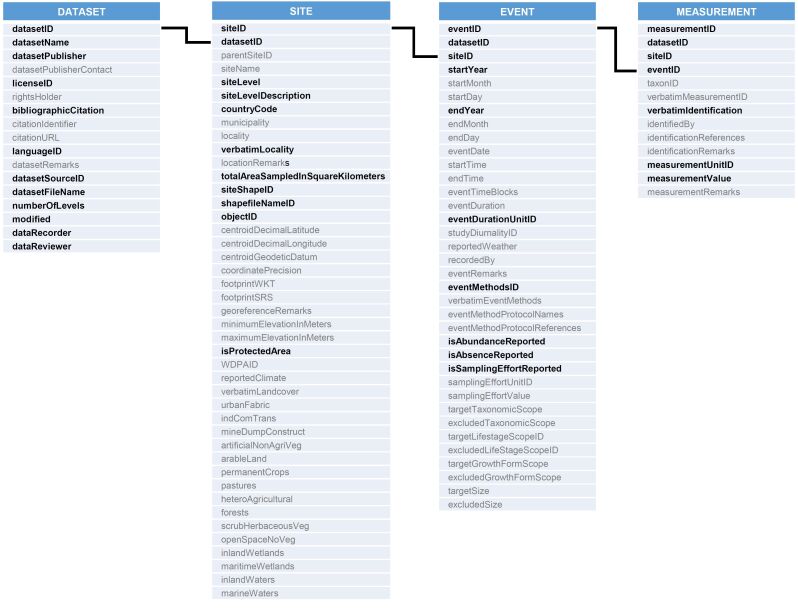
Graphical representation of the structure of the SPARSE database, with the four central tables (indicated by numbers). Fields for which data must be entered (i.e. required fields) are marked by bold black letters. Fields that are optional are marked by regular grey letters.

**MS Access file.** The main body of SPARSE in the *SPARSE.accdb* file is implemented in four tables (Fig. 2):

1. **DATASET** table in which each row is a publication (or an online published dataset) that contains information on authors, year of publication, language, accessibility and personnel who mobilised the data to SPARSE. Each dataset can contain one or more sites, each with one or more sampling events.
2. **SITE** table in which each row is a site that was surveyed for species (during a sampling event). One site can be surveyed more than once, i.e. can have multiple sampling events, which then form a time series. The table contains information on geographic location, area, unique *objectID* of a corresponding spatial object (point, line, polygon) in three ESRI shapefiles (for points, lines and polygons), as well as protection status and land cover.
3. **EVENT** table in which each row is a temporally well-defined sampling event done at a particular site. The table contains information on time, duration, sampling effort, method and taxonomic scope of events.
4. **MEASUREMENT** table in which each row is an observation of a particular species during a particular event. The table contains information on the unit of abundance or incidence that was recorded (e.g. abundance, density, percentage cover, presence/absence) and on taxonomic details of each species.

Each table comes with several **codebooks** (CB) which list predefined controlled vocabulary for selected fields. These can be re-defined, or removed, by users according to their specific needs.

**Field definitions.** Detailed description of each field in these four tables is provided in the DEFINITIONS table in the *SPARSE_definitions.xlsx* file. The most important columns in the table are:

- **Data type**: Describes the type of stored data, for example, a number, text etc.
- **Required field**: Is the information required (R) or optional (-)? The distinction between these two categories allows for easier data input in case the information in the original publications is limited and the optional fields can, thus, be skipped. In contrast, the required fields are essential, sometimes even critical for the integrity of the whole database.
- **Controlled vocabulary**: The field relies on a codebook (CB) that provides predefined categories or it does not (-).
- **Duplicity allowed**: The datum in the field must have a unique value (ND) or it is allowed to be a duplicated value (-).
- **Source of the definition**: We took the definition from Darwin core (DWC, Wieczorek et al. 2012), Humboldt core (HC, Guralnick et al. 2017); alternatively, we came up with our own definition (SPARSE) or the field is a primary integer key (key).
- **Column name**: Name of the field, as in a corresponding MS Access table.
- **Column description**: Detailed definition of the field, sometimes with examples.

**Shapefiles.** Each site in the SITE table is associated with a point, line or polygon geometry (through a combination of *siteShapeID* and *objectID*) that are stored in three shapefiles in the *SPARSE_shapefiles* folder.

**Bibliographic information.** Each dataset in the DATASET table comes with a detailed bibliographic reference stored in a *SPARSE_bibliography.bib* BibTeX file. This is to facilitate citations of the original publications from which we extracted the data. See the *Re-use potential and licensing* section below for details.

### Example data: Czech birds

We provide an example use of the SPARSE framework by digitising data on birds from the Czech Republic, from 348 sites and 524 sampling events, with 15,969 unique species-per-event observations of presence, abundance or population density.

The **data extraction and input procedure**, step-by-step, was as follows:

- **Online search.** We searched for inventory studies mentioned in Storch and Kotecky (1999) and in the Czech NDOP (https://portal.nature.cz/nd/) and DRUSOP databases (https://drusop.nature.cz/portal/). Studies in these sources had partly been digitised in a spreadsheet form, i.e. usually the site-by-occurrence tables were available, but we still had to extract the metadata on methods, habitats and spatio-temporal settings of the studies. We also conducted an extensive online search in Google, Google Scholar and other search engines and downloaded all the freely available .pdf files that we found. These were mostly unavailable in a digitised spreadsheet form.
- **Conversion of .pdf files to .xlsx template.** We transcribed or copied the relevant parts from the .pdfs to an .xlsx template file *INPUT_data-template.xlsx*. Many of the studies are scanned documents from which the data cannot be readily copy-pasted and we transcribed these manually. The template also contains checking procedures and macros that ensure that the data are input correctly.
- **Import to MS Access.** We imported the data from the .xlsx files to the .accdb database (in MS Access: External Data -> New Data Source -> From file -> Excel). Importantly, we made sure that the IDs linking individual tables to each other did not duplicate IDs already in the database.
- **Geolocation.** In a parallel effort, we geolocated the sites and we manually converted them into points, lines or polygons using ESRI ArcGIS Desktop 10.0.1.

These are the references from which we have extracted data to SPARSE 1.0: Klíma 1959, Vlček 1969, Vlček 1971, Vlček 1973a, Vlček 1973b, Vlček 1974a, Vlček 1974b, Rybář 1975, Pecl 1975, Vlček 1977, Hudec 1975, Vlček 1975, Rybář 1976, Vlček 1976, Šťastný 1978, Sofron et al. 1978, Tis - Svaz pro ochranu přírody, krajiny a lidí 1976, Šťastný et al. 1987, Hlaváč and Hříbek 1985, Šmaha 1984, Řepa 1979, Řepa 1980a, Zikmundová 1976, Šmaha 1978, Tichý 1981, Tichý 1985, Řepa 1981, Řepa 1986, Šmaha 1983, Řepa 1980b, Kovářová 1984, Bureš and Maton 1985, Lemberk 1989, Bürger and Kloubec 1991, Pavelka 1988, Vlček 1983, Pelc 1989, Pešková 1990, Palička and Kopec 1993, Bárta 1986, Tichý 1992, Pokorný 1989, Lemberk 1996, Kučera 1998, Kloubec and Klimeš 1995, Pokorný 1990, Bürger and Kloubec 1996, Poprach and Vrbková 2014, Berec 1993, Lemberk and Fejfar 1995, Francová 1990, Kloubec 1996, Bejček and Tyrner 1980, Poprach et al. 2014, Ševčík 1994, Lemberk and Růžička 1996, Berec and Pavelka 1993, Pykal 1991, Flousek 1994, Klimeš 1994, Hanák 1996, Honza 1992, Řepa 1984, Řepa 1989, Bárta 2000, Zasadil 2001, Řepa 1994, Lemberk 1994, Jansová 1993, Kočvara 2005, Dušek 2004, Stříteský and Krist 2004, Macháček 1997, Ševčíková and Tošenovský 2014, Pearce and Boyce 2006, Kočvara 2004, Paclík et al. 2003, Kočvara 2006, Kočvara 2007, Tichai 2012, Spolek pro ochranu mokřadů (SPOM) 2006a, Křenek 2006b, Šebestian 2008, Bárta 2014, Křenek 2006a, Foit 2013, Fischer and Vlach 2012, Hanák 2006, Kočvara 2014, Urbánek 2012, Doupal and Hajný 2007, Pokorný 2014, Řepa 2014, Růžička 2010, Machar 2007, Spolek pro ochranu mokřadů (SPOM) 2006b, Honců 2013, Chytil 2013, Řepa 2016, Machar 2009, Řepa 2015, Pokorný 2013, Černý and Šebela 2018, Řepa 2017, König et al. 2019, Řepa 2018, Volf 2019, Růžička 2020.

Together, these data have the following taxonomic, temporal and geographic coverage:

**Taxonomic coverage**: The dataset covers birds (Vertebrata, Aves), using the IOC World Bird List (v. 11.1) taxonomy of Gill et al. (2021).

**Figure 3.**
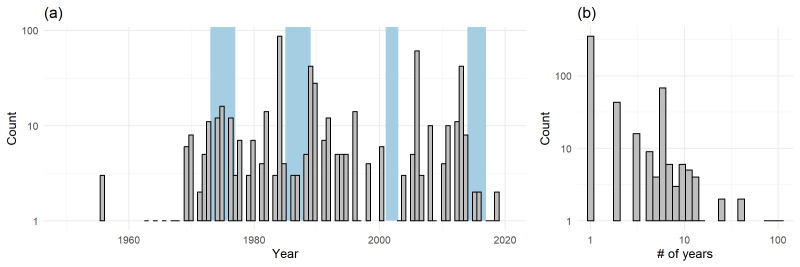
(a) The temporal extent of the dataset; the histogram summarises starting sampling years of all events (log_10_ y axis). Blue rectangles represent the four temporal periods covered by the Czech Breeding Bird Atlases (Hudec et al. 1987, Šťastný et al. 1997, Bejček et al. 2006, Šťastný et al. 2021); (b) Temporal durations of events in the dataset (log_10_ x and y axes).

**Temporal coverage**: The dataset covers the period between 1890 and 2020. Some inventories use very old data, mostly haphazard/non-systematic observations, sometimes from local people. The oldest start year of "event" survey is 1890 (e.g. Pelc 1989, Kloubec and Klimeš 1995). However, most of the inventories were made after 1960 (Fig. 3).

**Figure 4.**
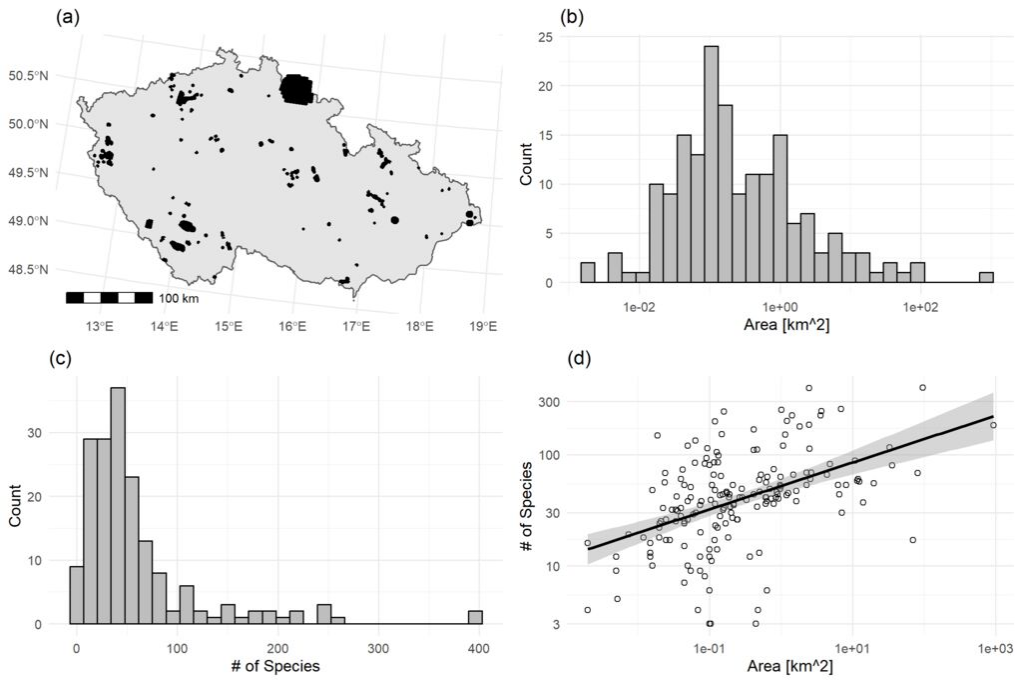
(a) Map of sites at which inventories were done. These represent a mix of point, line and polygon objects; (b) Histogram of areas the sites (log_10_ x axis); (c) Histogram of number of species detected at each site (log_10_ x axis); (d) Relationship between area of each site and number of species detected at the site (log_10_ x and y axes), with a linear regression fitted through the data. Grey band indicates standard errors.

**Geographic coverage**: The bird dataset covers the area of the Czech Republic, Europe (Fig. 4), with the bounding box delineated by 48.55 and 51.06 Latitude and 18.87 and 12.09 Longitude.

## Data resources

A dynamic GitHub repository of SPARSE, which may undergo development and updates in the future, is at https://github.com/petrkeil/SPARSE1.

A static fixed version of the database in a .zip file, which contains all the files as on the date of submission (25 May 2023), is provided as Suppl. material 1 with this paper.

## Re-use potential and licensing

**Applicability to other taxa.** We envision that SPARSE can be re-used for taxa other than birds, as well as for other geographic areas. The main properties that facilitate this cross-taxon use are: (1) The flexible nature of the *measurementUnitID* field (in the MEASUREMENT table), which can accommodate presences/absences, abundances, but also densities, estimates of percentage cover (e.g. in botanical plots), biomass etc.; (2) flexible taxonomic metadata (in the MEASUREMENT and EVENT tables); (3) flexible metadata on methods (in the EVENT table), that can cover a range of methods, from ornithological point counts to entomological sweeping net surveys or botanical relevees.

**Interoperability and openness.** SPARSE currently uses .xlsx and .accdb file formats, which may reduce interoperability when compared to, for example, .csv files, but they can still be opened using free and open software. Specifically, the **.xlsx** file format is an Office Open XML (developed by Microsoft), it is fully open (see here https://en.wikipedia.org/wiki/Office_Open_XML) and users can open any .xlsx file in common free and open programmes, such as Libre Office Calc. The **.accdb** file can be opened using free and open tools, such as MDB Tools, Jakcess or Libre Office Base. As a result of this, we consider the data as *de facto* open. However, particularly the decision to use the .accdb file was not taken lightly; the initial versions of SPARSE were implemented in Libre Office Base. The decision to migrate to .accdb was made because, at the time of implementation, MS Access was more user-friendly, the authors were familiar with it and we had access to specific training that was unavailable for Libre Office Base. In addition, SPARSE also uses **ESRI shapefiles**. Similarly to .xlsx, this format has been developed by a commercial company (ESRI), but the format itself is fully open and can be imported to free and open software, such as R or QGIS.

**Licensing and attribution.** We have put the SPARSE database structure under the **Creative Commons Attribution (CC-BY) license 4.0** (https://creativecommons.org/licenses/by/4.0). Users of the SPARSE framework, or those who modify it, should cite this publication. Users of the Czech bird data that accompany this publication should cite the original publications (studies) in which the inventories were first published. If they take the data from SPARSE (as opposed to directly extracting it from the original publications), we would like to ask them to also cite this publication. A BibTeX file with all the references accompanies the raw data and can be loaded to common reference managers, such as Zotero or JabRef.

## Supplementary Material

9858DCB5-5B9F-55F1-80AE-B64B850594C5 10.3897/BDJ.11.e108731.suppl1 Supplementary material 1 SPARSE 1.0 - all database files as on the date of submission Data type all occurrence data, metadata, bibliography, shapefiles and associated R code Brief description This .zip archive contains the SPARSE 1.0 database as on the date of submission of the manuscript (22 June 2023). File: oo_926169.zip https://binary.pensoft.net/file/926169 Petr Keil, Eva Trávníčková, Florencia Grattarola, Clara Rosse, Kateřina Tschernosterová
