# Supplementary material for: SPARSE 1.0: a template for databases of species inventories, with an open example of Czech birds
Source: Biodivers Data J. 2023 Nov 23;11:e108731. doi: 10.3897/BDJ.11.e108731 (PMC10690794; doi:10.3897/BDJ.11.e108731)
Supplement: Supplementary material 1 — SPARSE 1.0 - all database files as on the date of submission [file bdj-11-e108731-s001.zip › INPUT_data-example_original_Repa_1980.pdf]

Kramerius 5

Digitální knihovna

Podmínky využití

Knihovna AV ČR poskytuje přístup k digitalizovaným dokumentům pouze pro nekomerční, vědecké a studijní účely a pouze pro osobní potřebu uživatelů. Část dokumentů Digitální knihovny AV ČR podléhá autorským právům. Využitím digitální knihovny a vygenerování kopie části digitalizovaného dokumentu se uživatel zavazuje dodržovat tyto podmínky, které musí být součástí každé zhotovené kopie. Jakékoli další kopírování materiálů z digitální knihovny není možné bez případného písemného svolení Knihovny AV ČR.

Hlavní název: **Folia zoologica. Monographs**
Vydavatel: **Academia**
Vydáváno v letech: **[1977-]**
Číslo ročníku: **29**
Datum vydání čísla: **1980**
Identifikátor ISSN: **0139-7893**
Stránky: **171, 172, 173, 174, 175, 176, 177, 178, 179, 180, 181, 182, 183, 184**

FOLIA ZOOLOGICA — 29(2): 171—184 (1980)

THE BIRD SYNUSIA OF TWO VILLAGES IN THE ČESKÝ LES MOUNTAINS (SOUTHWESTERN BOHEMIA)

СООБЩЕСТВО ПТИЦ В ДВУХ НАСЕЛЕННЫХ ПУНКТАХ РАСПОЛОЖЕННЫХ В ГОРАХ ЧЕШСКОЙ ЛЕС (ЮГОЗАПАДНАЯ ЧЕХИЯ)

Pavel ŘEPA Received August 31, 1978

District Museum at Tachov

Abstract

In the years 1974—1976, the author investigated the composition of both breeding (by mapping of breeding territories) and non-breeding (by census in transects) synusia of birds in two villages situated in the lower part of the Český les Mountains, in a region where woodland predominates over open landscape. One village (Kateřina) is closely surrounded by fields, the other one (Diana), by a forest.

The total density of the breeding synusium of birds in the course of three years of investigation amounted to 9.6 pairs per 1 hectare on the average in the first-mentioned village, and to 15.2 pairs per 1 hectare in the other one. There were 34 and 32 species of breeding birds respectively. The diversity of the breeding synusium was rather high, its values being 2.94 in the village Kateřina and 2.79 in the village Diana. In both villages the following species were dominant in the breeding synusium (over 5 % of the total numbers of breeding pairs): *Passer montanus, Fringilla coelebs, Delichon urbica* and *Carduelis chloris. Hirundo rustica* was dominant only at Kateřina; *Turdus merula* and *Parus major*, only at Diana.

In the non-breeding periods 52 species were ascertained at Diana and 63 species at Kateřina. The density of bird synusia fluctuated, at Kateřina, between 67 individuals per 10 hectares in winter and 398 individuals per 10 hectares in late summer; at Diana, between 133 individuals per 10 hectares in winter and 339 individuals per 10 hectares in late summer. In both villages *Passer domesticus* and *Carduelis chloris* were dominant all the year round. *Parus major, P. caeruleus, Hirundo rustica, Delichon urbica, Carduelis carduelis* and *Emberiza citrinella* rearched, in both villages, the rank of dominant species at least in some non-breeding periods. *Pyrrhula pyrrhula* and *Sitta europaea* belonged to the dominant species only at Diana during winter.

Introduction

The topic of the present paper is a survey of results of three years' investigations on bird synusia in two villages situated in a kettle valley called Kateřinská kotlina in the Český les Mountains (southwestern Bohemia, district of Tachov). In the district of Tachov, during the last two decades an extraordinarily intensive advancement of agriculture has taken place, which is connected with a conspicuous increase in, and consolidation of, land used for agriculture. It results in a decline in the quantity of dispersed non-forest woody stands in the landscape in the course of relatively short time. At the same time, agricultural production establishments dissappeared from the village, because the production has changed to large-scale ways of production (cattle breeding, machinery and tractor stations). The large-scale agricultural plants are usually localized outside the proper built-up area of the villages. This phenomenon, along with the advancing planting-out of woody plants in the villages, makes it possible, for birds

adapted to small tree complexes, to use the green plots of villages as refuges.

Our knowledge of the aviafauna of human habitations is mostly limited to urban habitats (see the extensive list of references in Hudec 1976); much less attention has been paid to the village habitat. From Czechoslovakia only some data are to be found in the papers by Hudec (1973) and Havlín (1975) and some data on the avifauna of Slovakian villages are included in the landscape-ecological analyses by Brtek & Feriancová-Masárová (1969), Feriancová-Masárová (1963, 1968), Turček (1958, 1971) and Ferianc (1967, 1968). Analogous is the situation in other European countries. For example, from Germany I gathered several fragmentary data on the bird population of lonely farmsteads in the papers by Dircksen & Höhner (1963) and Mildenberger (1950). Only in England were the investigations of avifauna in the village habitat carried out intensively (*e.g.*, Benson & Williamson 1972); however, the investigations pertain to isolated farmsteads, *i.e.*, a habitat type which is quite different from our villages.

I decided therefore to make a series of investigations in various village types with the aim to obtain knowledge of the possibilities to make use of villages as refuges for the occurrence of birds in an open agricultural landscape. At first I chose, for my investigation, villages situated in a region which, owing to its submontane character, has not yet been used for agriculture very intensively. It concerns the villages Kateřina and Diana, lying between the towns Přimda and Rozvadov in the Český les Mountains. Their elevation and climate (in the rough) conform to the conditions in the region of the most intensive agricultural activity in the submontane area of the Český les Mts.; however, they lie in an area of agricultural landscape which is surrounded on all sides by an extensive forest complex.

I wish to thank RNDr. Karel Hudec, CSc., Institute of Vertebrate Zoology, Czechoslovak Academy of Sciences, in Brno, for critical reading of a draft of this paper.

Description of the Investigated Villages

Village Kateřina – (250 inhabitants – a type of village closely surrounded by fields. It lies in an area of open landscape, about 10 × 10 km in size, along the Kateřinský potok Brook, not far from the township of Rozvadov (see Fig. 1), at a distance of about 1 km from the nearest forest complex. Its elevation is 510–520 m a.s.l.

The village extends along a road about 1 km in length (Fig. 2); it consists of one-storeyed village houses with small gardens. There are no functioning buildings of agricultural production within the village. 30 years ago, many buildings of the village were abandoned; all of them have already been demolished and their rests have been overgrown with shrubs and trees. The green plots in the village are represented by orchards which are often desolated, with superannuated trees; furthermore, there are scattered maples, elms and lime-trees along the road; locally, also thickets of young trees and shrubs arose by natural reproduction in the ruins. On the western side the village adjoins the bed of the Kateřinský potok Brook which is bordered by a broad swamp. The proper brook bed is bordered by an old dense alder wood which adjoins, in the middle part of the village, the built-

-up area and contributes to the verdure of the village. In the northern part of the village the built-up area is interrupted by the bed of the Rybniční potok Brook (a left tributary to the Kateřinský potok Brook), so that the road passing the centre of the village is surrounded by a reed swamp about 100 m broad; this swamp secludes the proper village from the hamlet Kateřinské Chalupy. Here also a small pine forest grows. Over the swamp, to the west of the village, cultivated fields extend. The fields also surround the village on all other sides.

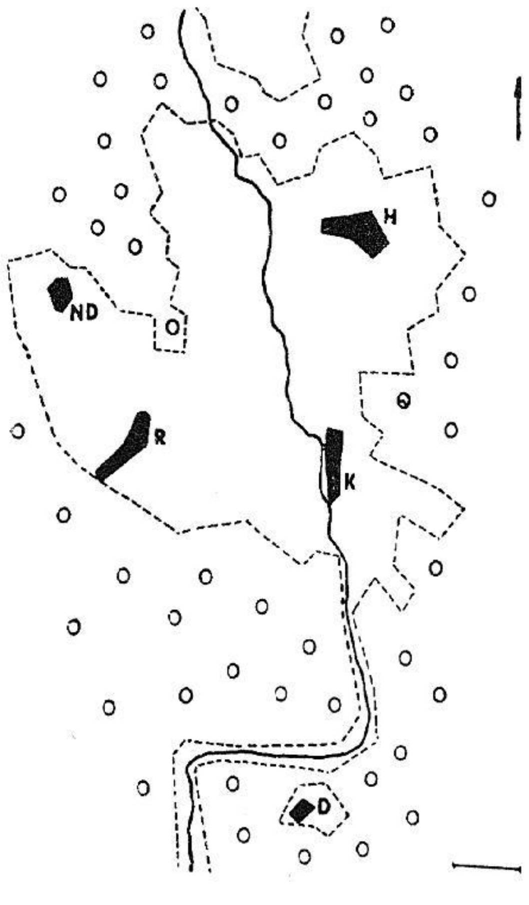

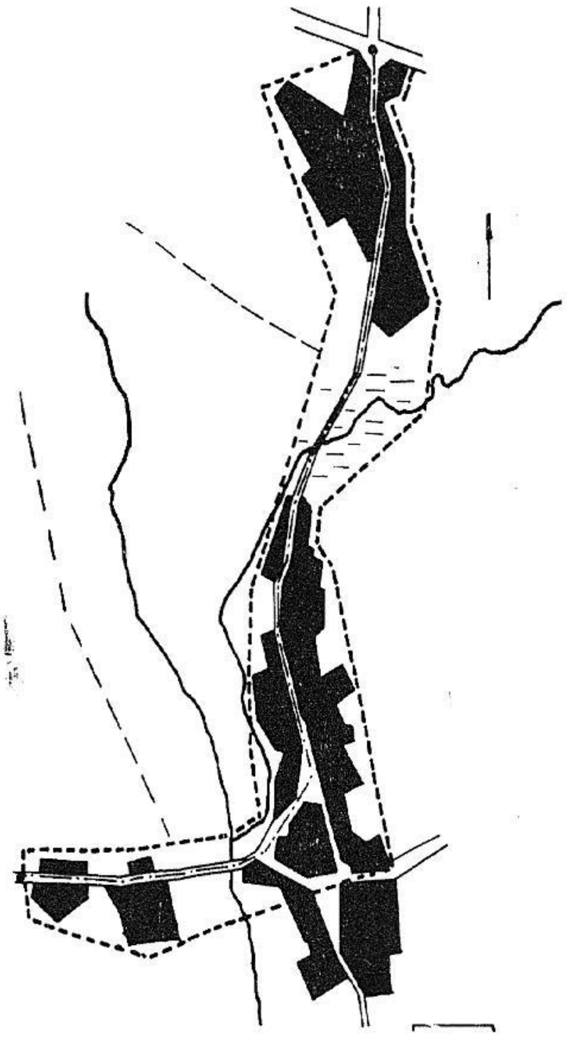

Fig. 1 (left). Schematic map of the kettle valley Kateřinská kotlina in the Český les Mts., showing the situation of the villages under investigation with respect to the forest complexes. The borders of the forest complexes in comparison with reality, are simplified. Explanations: dark spots, villages (H, Hoštka; ND, Nové Domky; R, Rozvadov; K, Kateřina; D, Diana); heavy solid line, course of the Kateřinský potok dashed fine lines, borders of the forest complexes; areas dotted with small circles, forest complexes. The abscissa in the lower right-hand corner indicates the length of 1 km; the arrow in the upper right-hand corner points to the north.

Fig. 2 (right). Schematic plan of the village Kateřina. Explanations: the dark spots represents blocks of village houses and back-gardens; the heavy dashed line marks off the area of investigation and simultaneously represents the border of the inside part of the village, outside of which – except the part limited by the fine dashed line – lie the fields; the area limited by the fine dashed line represents the swamp adjacent to the Kateřinský potok Brook; the dashing inside the village indicates the place where the swamp penetrates into the village the fine dot-and-dash line indicates the transect axis of the census in the non-breeding periods.

The village Diana (cca 300 inhabitans) represents a type of a village surrounded immediately by the forest (Fig. 3).

It is situated in the centre of a small area of fields and meadows, which, on all sides, is surrounded by continuous forests. The nearest distance from the edge of the forest complex is 4—5 km. The elevation is 540 m a.s.l. The village also extends along a road, only in the centre there is a stronger agglomeration of buildings. The village houses are scattered; among the gardens are large areas overgrown with grass or with old trees. Along the road, on both sides, there is an avenue of old horse-chestnut trees. In the south, a desolate English park with a manor-house is contiguous with the village. On the other sides, meadows are closely contiguous with the village; in one place there is also a forest nursery. The forest edges lie at most 150 m far from the village outskirts. There are no agricultural of farming objects, only a stable with several horses of the Forest Establishment is in use there.

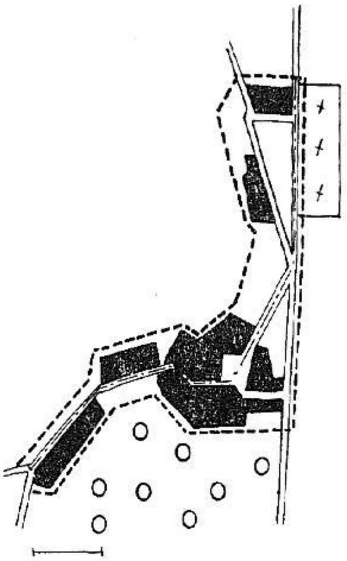

Fig. 3. Schematic plan of the village Diana. Explanations as in Fig. 2. The village edges with meadows; small crosses mark the forest nursery and small circles the area of the park.

Methods

The bird census during the breeding period was carried out by mapping the breeding territories (E n e m a r 1959). In the period from April 15 to June 15, 1974—1976, 6—8 cheks were carried out respectively. It is a minimum number which can involve many inaccuracies and errors (B e r t h o l d 1976). Therefore I paid extraordinary attention to precising my results by seeking nests; information obtained from local inhabitans, especially from the forest staff, was very helpful to me in this respect. In the case of the House Sparrow and Tree Sparrow, Starling, Swallow and House Martin I determined the number of breeding pairs on the basis of the number of nests only. In other species I could confirm the occurrence of many pairs by finding their nests. In the case of the thrush-like birds, Chaffinch and Greenfinch, subsequent seeking of nests after leaf fall proved to be advantageous (T u r č e k 1956). I also made use of some instructions for precising the mapping of the breeding territories within the urban habitat, as given by T o m i a l o j c (1968) and L e n z (1971).

The breeding territories in the village Kateřina were mapped almost all over the territory of the village (only a part of the southern end of the village was left out owing to the lack of time) in an area of 11,74 hectares. In the village Diana the whole territory, except the manor park, was investigated (area 6,36 hectares).

During the non-breeding periods, the census was made on standard transects (N o v i k o v 1953) which were laid through the centre of the

villages along the roads. The breadth of the transects was 50 m; their length was 1180 m in Kateřina and 900 m in Diana, so that density data were converted to 5.9 hectares in Kateřina and 4.5 hectares in Diana. The censuses were made regularly twice a month all over the rest of the years 1974—1976 (from January 1 to April 15 and from June 15 to December 31). The year was divided into 5 periods: I, spring (March and first half of April); II, post-breeding (second half of June, July); III, late summer (August and September); IV, autumn (October and November); and V, winter (January, February and December). The data from each period were pooled for all three years. I calculated the density (individuals per 10 hectares) and dominance (Palmgren 1930). For a comparison of both villages, the values of Sørensen's index (according to the formula $\frac{2c}{a+b}$, where a and b are numbers of species in first and second synusium, c is the number of species common to both synusia) and Renkonen's number (the sum of smaller values of dominance from both compared synusia) were calculated (Pikula 1976). Furthermore, according to the Shannon—Weawer's formula $D = -\sum P_i \,.\, lnP_i$, where $P_i = \frac{n}{N}$, $n =$ = number of individuals of a species in the synusium, N = number of all individuals in the synusium) the diversity and according the Pielou's formula ($H = D/lnS$, S = number of species in the synusium) the species evenness were calculated (Odum 1977). The species were divided into dominant, influent and accessory by their dominance (Palmgren 1930).

Results

The results of the investigation of breeding synusia are summarized in Tab. 1. The density of breeding birds was distinctly higher at Diana in successive years as well as on the three years' average, whereas the number of breeding species was about the same in both villages. From Tab. 2, showing the density and dominance of individual ecological groups according to the way of breeding, it is evident that — apart from the ground-breeding species — a higher density in all groups was found in the village Diana. It may be due to the smaller area of this village. Then it would be clear that the rule of higher density in smaller plots of little field groves (Peitzmeier 1950) is of general validity for any small area of a certain habitat which is fully surrounded by another type of habitat, in our case, for the villages. An extraordinary increase in the density at Diana, in comparison with Kateřina, was shown by species breeding in hollows and on buildings. The increase in the number of hollow-breeding species is due to the higher number of hollow trees at Diana (an old avenue); the increase in the number of species breeding on buildings is due to *Delichon urbica* which, at Diana, is breeding in a large number on a stable. No livestock rearing is carried out at Kateřina.

The fluctuation in the density of the breeding synusia during successive years was not very great in both villages. The villages were not very different either as regards the qualitative composition of the breeding synusia in successive years. The stable species, breeding in the course of all three

years, represented 65.5 % of the total species number at Diana, and 67.5 % at Kateřina (Tab. 3).

The species diversity of the community and the species evenness, which indicates the balance of the community, are relatively high in both villages; at Kateřina they were somewhat higher than at Diana.

Fig. 4 shows the pyramids of numbers according to Evans, indicating the significance of dominant, influent and accessory species in the synusium. The pyramid is more favourable for Kateřina, indicating the minor significance of the dominant and a very significant share of the influent species. At Diana, the share of the dominant species was much more significant owing to the extraordinarily numerous occurrence of *Delichon urbica.*

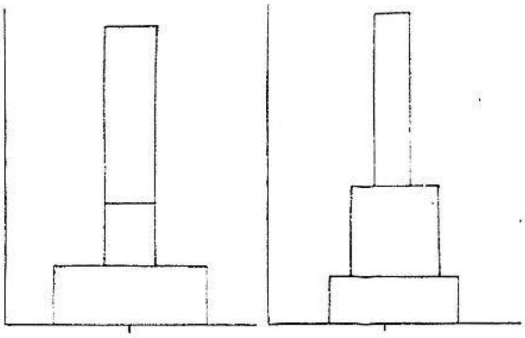

Fig. 4. The pyramids of numbers after Evans. Left, village Diana; right, village Kateřina. Explanations: absissa, abundance in per cent of the total number of pairs; ordinate, number of species in per cent of the total number of species present; the lower rectangle corresponds to dominant, the middle one to influent and the upper one to the accessory species.

The similarity of breeding synusia in both villages is rather high (Sørensen's index 79.0; Renkonen's number 70.5). 26 breeding species are common to both villages, all dominant species belonging to them. Contrary to Kateřina, the forest species, especially the hollow-breeding ones, mainly occur at Diana (the influent *Sitta europaea* and the accessory species *Certhia familiaris, Ficedula hypoleuca, Sylvia atricapilla, S. borin, Aegithalos caudatus*). At Kateřina, all additional breeding species (contrary to Diana) were accessory. It concerned the shrub-dwelling species *(Lanius collurio, Sylvia communis, Phylloscopus trochilus, Troglodytes troglodytes)* and the species bound to the neighbourhood of water and to reed stands *(Motacilla cinerea, Acrocephalus scirpaceus, Emberiza schoeniclus)* and one climber species *(Iynx torquilla)* as well. Of the species common to both villages, the greatest difference of dominance and density of occurrence were found for *Delichon urbica* which, apparently owing to the existence of a horse-stable, was much more frequent at Diana; furthermore for *Passer domesticus*, the dominance of which in the village surrounded by a forest (Diana) was a half lower than in the village surrounded by fields (Kateřina). The influence of better food conditions in the field village apparently plays a role here. Distinct was the higher share of the hollow-breeders, especially of *Parus major* and *Sitta europaea* at Diana. The

Explanations to Tab. 1.:

Non-breeding species ascertained during the breeding period:

Kateřina: *Streptopelia turtur, Cuculus canorus, Aegithalos caudatus, Sitta europaea, Certhia familiaris, Locustella fluviatilis, L. naevia, Motacilla flava, Carduelis spinus, Pyrrhula pyrrhula.*

Diana: *Iynx torquilla, Lanius excubitor, Parus ater, Phylloscopus sibilatrix, Muscicapa striata, Pyrrhula pyrrhula.*

Explanations: First number, Kateřina; second number, Diana

Tab. 1. Composition of the breeding synusia of the villages Diana and Kateřina in 1974—1976

Species	Number of breeding pairs			Mean values	Density, pairs per 1 hectare 1974—1976	Dominance 1974—1976
	1974	1975	1976			
Streptopelia decaocto	–/1	1/–	1/–	0.7/0.3	0.06/0.05	0.6/0.3
Dendrocopos major	–/1	1/1	–/1	0.3/1.0	0.03/0.16	0.3/1,0
Iynx torquilla	–/–	1/–	1/–	0.7/–	0.06/–	0.6/–
Sturnus vulgaris	1/2	1/3	1/2	1.0/2.3	0.08/0.36	2.4/0.9
Parus major	5/7	5/8	5/7	5.0/7.3	0.42/1.15	4.4/7.5
Parus caeruleus	2/2	2/2	2/2	2.0/2.0	0.17/0.31	1.8/2.0
Parus palustris	1/1	–/–	1/1	0.7/0.7	0.06/0.10	0.6/0.7
Aegithalos caudatus	–/1	–/–	–/–	–/0.3	–/0.05	–/0.3
Lanius collurio	–/–	1/–	–/–	0.3/–	0.07/–	0.3/-
Sitta europaea	–/4	–/6	–/4	–/4.7	–/0.73	–/4.8
Certhia familiaris	–/1	–/2	–/1	–/1.3	–/0.21	–/1.4
Troglodytes troglodytes	–/–	1/–	1/–	0.7/–	0.6/–	0.6/–
Prunella modularis	–/–	–/1	1/1	0.7/0.3	0.06/0.05	0.6/0.3
Turdus merula	5/5	5/6	5/4	5.0/5.0	0.42/0.79	4.4/5.1
Turdus philomelos	3/5	2/3	5/4	3.3/4.0	0.28/0.63	2.9/4.1
Turdus pilaris	4/2	4/1	2/1	3.3/1.3	0.28/0.21	2.9/1.4
Erithacus rubecula	1/1	–/2	2/2	1.7/1.0	0.14/0.16	1.5/1.0
Phoenicurus ochruros	1/1	1/1	2/1	1.3/1.0	0.11/0.16	1.2/1.0
Phoenicurus phoenicurus	1/1	–/1	1/1	1.0/0.7	0.08/0.10	0.9/0.7
Sylvia atricapilla	–/–	–/1	–/1	–/0.7	–/0.10	–/0.7
Sylvia borin	–/1	–/–	–/1	–/0.7	–/0.10	–/0.7
Sylvia curruca	2/1	3/–	2/1	2,3/0.7	0.20/0.10	2.0/0.7
Sylvia communis	1/–	2/–	1/–	1.3/–	0.11/–	1.2/–
Phylloscopus collybita	4/1	2/1	1/1	2.3/1.0	0.20/0.16	2.0/1.0
Phylloscopus trochilus	5/–	3/–	3/–	3.7/–	0.31/–	3.2/–
Acrocephalus scirpaceus	1/–	1/–	–/–	0.7/–	0.06/–	0.6/–
Ficedula hypoleuca	–/1	–/1	–/–	–/0.7	–/0.10	–/0.7
Motacilla alba	2/2	1/1	1/1	1.3/1.3	0.11/0.21	1.2/1.4
Hirundo rustica	7/5	6/4	4/3	5.7/4.0	0.48/0.63	5.0/4.1
Delichon urbica	6/21	8/15	9/16	7.7/17.3	0.65/2.72	6.7/18.0
Carduelis chloris	8/6	10/7	10/8	9.3/7.0	0.79/1.10	8.2/7.2
Carduelis carduelis	6/2	3/1	4/1	4.3/1.7	0.37/0.26	3.8/1.7
Carduelis cannabina	–/1	1/–	1/1	0.7/0.7	0.06/0.10	0.6/0.7
Serinus serinus	4/1	5/2	4/4	4.3/2.3	0.37/0.36	3.8/2.4
Fringilla coelebs	13/13	15/14	14/13	12.3/13.3	1.19/2.09	12.2/13.7
Emberiza citrinella	6/1	3/3	6/1	5.0/1.7	0.42/0.26	4.4/1.7
Emberiza schoeniclus	1/–	1/–	–/–	0.7/–	0.06/–	0.6/–
Passer montanus	3/1	4/2	3/2	3.3/1.7	0.28/0.26	2.9/1.7
Passer domesticus	19/8	20/9	19/10	19.3/9.0	1.65/1.41	17.0/9.3
Total number of species	26<br>30	32<br>23	29<br>29	34<br>32		
Total number of pairs	112<br>100	117<br>94	112<br>97	113.5<br>97.0		
Total density, pairs per 1 hectare	9.47<br>15.73	9.89<br>14.81	9.55<br>15.32	9.65<br>15.21		
Species diversity of the synusium	2.89<br>2.90	2.89<br>2.73	2.87<br>2.87	2.94<br>2.79		
Evenness	0.90<br>0.83	0.90<br>0.85	0.86<br>0.87	0.82<br>0.80		

higher representation of the grain-eating species, *Carduelis carduelis*, and *Emberiza citrinella*, in the field-surrounded village Kateřina should also be mentioned.

A survey of the composition of the bird synusia (dominant species only) in the course of the year is given in Tab. 4 and 5. The highest density in both villages occurred in late summer; only a little lower density in the post-breeding period. During the passage periods, a higher density was ascertained at Diana in spring; in autumn, on the contrary, at Kateřina. In winter the density was distinctly the lowest at Kateřina, whereas at Diana it was only little lower than in autumn. The difference in the changes of density in the course of the year is due mainly to the circumstance that Diana is surrounded by woodland. That is why a rapid decline of bird density occurred in autumn; the grain-eating birds (*Fringilla coelebs, Embe-*

Tab. 2. Composition of the breeding bird synusia of both village under investigation according to the way of breeding. Mean values for 1974—1976

Breeding species	Kateřina			Diana		
	number of species	dominance in %	density, pairs per 1 ha	number of species	dominance in %	density, pairs 1 ha
Species breeding in hollows	9	13.6	1.29	11	23.9	3.64
Species breeding freely on trees	7	32.1	3.12	8	27.7	4.22
Species breeding in shrubs	11	19.0	1.84	8	13.6	2.09
Species breeding on ground	4	6.5	0.62	2	3.1	0.47
Species breeding on buildings	3	28.8	2.78	3	31.4	4.76

Tab. 3. Stability of the occurrence of breeding species in the course of three years of investigation in both villages

Breeding species	Diana		Kateřina	
	number of species	% of the total number	number of species	of % the total number
Species breeding in all three years	21	65.5	23	67.5
Species breeding in two years only	8	25.0	7	20.0
Species breeding in one year only	3	9.5	4	12.5

riza citrinella, Passer domesticus and *P. montanus, etc.*) left the wooded area and stayed in the fields. From the fields they rather frequently flew into the interior of the village Kateřina. On the contrary, at Diana a higher density was recorded in winter because the woodland birds sought food there.

The total number of species ascertained was the highest in both villages in the post-breeding period and in late summer. In spring and in autumn the number of species in both villages was distinctly lower and in winter it attained its minimum values. The number of species in all periods was a little higher at Kateřina than at Diana. In my opinion, it was due to the more diverse environment at Kateřina (the presence of a brook and a swamp, the structurally more diverse tree stands).

The similarity of the qualitative composition of bird synusia of both villages in successive non-breedings periods was identical with their similarity in the breeding period, sometimes it was even higher (the following are the values of Sørensen's indices for the successive periods: I, 74.0; II, 81.0; III, 83.0; IV, 68.0; V, 77.0). The similarity of both villages, as regards the quantitative composition of their bird synusia in the non-breeding periods, was a little lower than in the breeding period (value of Renkonen's numbers: I, 59.2; II, 78.8; III, 66.6; IV, 66.7; V, 69.5). It is evident that the highest similarity of the bird synusia of both villages was found during the breeding and post-breeding periods.

Tab. 4. Composition of the non-breeding synusia of birds in the village Kateřina. Mean values for 1974—1976. Dominant species only

Species	Period									
	Spring		Post-breeding		Late summer		Autumn		Winter	
	De	Do	De	Do	De	Do	De	Do	De	Do
Sturnus vulgaris	3.1	2.1	3.0	0.9	16.3	4.1	9.1	5.1	—	—
Parus major	17.4	11.9	10.9	3.2	22.9	5.7	18.6	10.6	15.2	22.1
Parus caeruleus	3.9	2.6	5.6	1.7	9.0	2.3	8.6	5.0	6.0	8.7
Turdus pilaris	4.5	3.1	3.6	1.0	1.9	0.5	4.6	2.6	3.4	5.0
Hirundo rustica	1.2	0.8	39.3	11.4	70.0	17.6	0.2	0.2	—	—
Delichon urbica	—	—	36.1	10.6	25.0	6.0	—	—	—	—
Carduelis chloris	22.5	15.4	35.8	10.4	46.8	11.8	21.8	11.8	4.5	6.7
Carduelis carduelis	0.5	0.3	23.5	6.9	22.7	5.7	5.0	2.9	2.2	3.3
Carduelis spinus	1.2	0.8	2.6	0.8	4.9	1.2	11.8	6.5	5.3	7.9
Fringilla coelebs	22.2	15.2	37.1	10.8	38.4	9.7	10.5	6.0	0.4	0.6
Emberiza citrinella	17.9	12.2	16.0	4.8	10.5	2.6	3.9	2.2	1.3	1.9
Passer domesticus	19.6	13.2	38.8	11.3	65.1	16.3	32.9	19.0	15.5	23.0
Passer montanus	5.8	3.9	13.8	3.9	7.1	1.8	7.4	4.3	0.7	1.0
Total density (ex. per 10 hectares)	147.0		342.2		398.0		171.3		67.7	
Total number of species	33		43		43		36		25	
Species diversity	2.42		2.73		2.69		2.80		2.31	
Evenness	0.69		0.70		0.69		0.78		0.72	

Explanations: De, density (number of individuals per 10 hectares); Do, dominance

Tab. 5. Composition of the non-breeding synusia of birds in the village Diana. Mean values for 1974—1976. Dominant species only

Species	Period									
	Spring		Post- breeding		Late summer		Autumn		Winter	
	De	Do	De	Do	De	Do	De	Do	De	Do
Parus major	37.3	15.1	10.4	4.0	18.2	5.3	26.2	21.2	43.0	23.0
Parus caeruleus	10.7	4.3	5.7	2.2	6.8	2.0	7.5	6.0	9.3	6.9
Sitta europaea	9.8	4.0	6.7	2.6	10.3	3.0	11.5	9.2	14.6	10.9
Delichon urbica	—	—	61.5	23.7	60.4	18.8	—	—	—	—
Carduelis chloris	16.4	6.7	31.6	12.1	28.0	8.3	8.0	6.5	4.3	3.3
Carduelis carduelis	—	—	13.3	5.1	19.9	2.7	1.0	0.8	—	—
Carduelis spinus	—	—	—	—	3.8	1.1	16.5	13.2	7.2	5.3
Pyrrhula pyrrhula	27.5	11.2	1.5	0.6	1.0	0.3	3.0	2.4	1.8	1.4
Fringilla coelebs	40.0	16.2	34.0	13.1	114.1	33.5	14.1	11.2	2.0	1.5
Fringilla montifringilla	25.3	10.3	—	—	—	—	—	—	0.7	0.5
Emberiza citrinella	0.4	0.2	5.2	2.0	5.6	1.7	2.8	2.3	9.6	7.2
Passer domesticus	24.2	9.9	25.4	9.9	23.4	6.9	15.7	12.7	33.7	17.2
Total density (ex. per 10 hectares)	245.6		260.4		339.2		124.9		133.7	
Total number of species	28		36		38		28		24	
Species diversity	2.39		2.57		2.09		2.53		2.18	
Evenness	0.71		0.72		0.56		0.75		0.68	

Explanations: De, density (number of individuals per 1 ha); Do, dominance'

The series of dominant species shows a number of common features in both villages in the non-breeding periods. In both villages, *Passer domesticus* was dominant all year round, although its share in the community of Diana was distinctly lower. Except in winter, *Fringilla coelebs* was dominant in both villages; *Carduelis chloris* was dominant at Kateřina in all seasons; at Diana in all seasons except the winter. In both villages, *Parus major* was dominant in all periods except the post-breeding one; in winter and in autumn also *Parus caeruleus* and *Carduelis spinus*. In the post-breeding period and in late summer, *Delichon urbica* was dominant in both villages, whereas *Hirundo rustica* at Kateřina only. *Emberiza citrinella* was dominant at Kateřina in spring but at Diana in winter. Further differences may be seen in the fact that *Pyrrhula pyrrhula* was dominant at Diana in spring and *Sitta europaea* in winter and in autumn; these species did not belong to the dominant species at Kateřina at all. Thus, the greatest differences in the dominant species of both villages were found in winter; the least ones, in the post-breeding period.

If the representation of the influent and accessory species in both villages in the non-breeding periods is compared, the following differences are found: During three years in the non-breeding periods, a total of 52 species were ascertained at Diana and 63 species at Kateřina. *Buteo buteo, Dendrocopos minor, Dryocopus martius, Columba palumbus, Nucifraga caryocatactes* and *Regulus regulus* were found only at Diana; *Phasianus*

colchicus, Cuculus canorus, Gallinago gallinago, Iynx torquilla, Parus montanus, P. cristatus, Saxicola rubetra, Acrocephalus scirpaceus, Locustella fluviatilis, Muscicapa striata, Loxia curvirostra and *Emberiza schoeniclus* were found only at Kateřina. All these species were ascertained only in one of the three years of investigation, usually also in one season only, and belonged to the accessory species. As a matter of fact, they were accidental visitors of the villages.

The stability of the occurrence in the course of three years of investigation during the non-breeding periods was higher at Kateřina, because of the species ascertained, 38 (60 %) were found in all three years. At Diana, only 26 (50 %) of the 52 species were ascertained in all three years.

The species diversity and evenness of the bird synusia in both villages were lower in the non-breeding than in the breeding periods. The difference between both villages was greater; in most non-breeding periods the diversity was distinctly higher at Kateřina. The lowest diversity in the course of the year was in winter and in spring in both villages, which was due to the strong predominance of dominant species in the winter synusia (*cf.* Pikula 1968). In the other seasons the diversity did not change very much at Kateřina; at Diana a rapid decline was observed at the end of summer due to a high share of *Fringilla coelebs* in the synusium at that time. *Fringilla coelebs* leaves the forest complexes at the end of summer, and big flocks gathered in the grassy areas of the village and its surroudings; they afterwards migrated into the open landscape. The species evenness did not change very much in the course of the year, only a slight decline was observed at Diana in late summer. An insignificant increase in the species evenness was observed at the time of the autumn migration.

Discussion and Conclusions

For a comparison with our data, the paper by Hudec (1973) dealing with two villages in the agricultural landscape in the environs of Brno, appears to be the most convenient. The paper by Havlín (1975) from the same region is based upon capture, not upon census data; the data available from Slovakia (Turček 1958, 1971, Ferianc 1967, 1968) are only qualitative.

The qualitative composition of the bird synusia of villages as given by Hudec (1973) resembles, by the total number of species found, our observations in the Český les Mountains. The representation of species is also rather similar, the differences pertaining exclusively to the less frequent species. In winter the synusia investigated by this author were dominated by *Corvus frugilegus* and *Corvus monedula*, which I did not observe in the village of the Český les Mountains at all.

Havlín (1975) found, in the village habitat, a similar number of species (50); the differences in the qualitative composition of the synusium pertained only the less frequent species.

The data by the Slovakian authors (Turček 1958, 1971, Ferianc 1967, 1968) do not differ by the number of ascertained species from our observations. Comparing the species composition of the bird synusia, howewer, more distinct differences are evident; many of them are of zoogeographical character.

A comparison of the quantitative composition of the village bird synusia

is possible only with the paper by H u d e c (1973). Conspicuous is the much lower share of *Passer domesticus* in the villages investigated by us. At the same time, in the village surrounded by forests (Diana), the share of *Passer domesticus* in the synusium is even lower than in the village surrounded by fields (Kateřina). Analogous is the situation of *Passer montanus*, the differences between the villages in the Český les Mts. and those in environs of Brno being not as distinct as for the species mentioned before. On the contrary, the share of *Fringilla coelebs*, *Parus major* and *Carduelis chloris* in the village in the woodland, investigated by us, was higher than in villages situated in an agricultural landscape (H u d e c 1973). Beyond doubt, these differences are due to the character of the landscape in which respective villages are situated. The villages in the Český les Mountains lie in a landscape in which woodland prevails over open country; those in the environs of Brno (H u d e c 1973), on the contrary, lie in a predominantly agricultural country. It seems therefore that the general character of the landscape influences the composition of the bird synusium more distinctly than the character of the nearest environs surrounding the village immediately. Hence, the differences between the villages in the Český les Mountains and in the environs of Brno, as regards the representation of some dominant species, are more distinct than the differences between the villages surrounded immediately by fields or forests and situated in the same region. However, the influence of the closest surroundings of a village is not negligible either, especially in the non-breeding periods. It is demonstrated by remarkable differences between the synusia of the villages investigated by us, as regards the overall density of birds, the frequency of their occurrence, the representation of some species, *etc.*

A more precise knowledge of the influence exerted by the whole region and by the immediate surroundings of a village on the composition of its bird synusia naturally requires further investigations of various village types.

Summary

(1) In 1974 through 1976, investigations of bird synusia were carried out in two villages situated in a kettle valley called Kateřinská kotlina in the Český les Mts. (southwestern Bohemia, district of Tachov). During the breeding periods, breeding territories were mapped; during the non-breeding periods censuses were made on transects. Both villages lie in a woodland area with less developed agriculture; one of them is closely adjacent to fields (Kateřina), the other one is surrounded by forests (Diana).

(2) In the village surrounded by forests the density of the breeding synusium amounted to 15.2 pairs per 1 hectare; 32 breeding species were ascertained, the species diversity was 2.79; their eveness, 0.80. The following species were dominant: *Passer domesticus*, *Fringilla coelebs*, *Delichon urbica*, *Carduelis chloris*, *Turdus merula* and *Parus major*. In the village surrounded by fields, the density of breeding synusium amounted to 9.6 pairs per 1 hectare; 34 breeding species were ascertained; the species diversity was 2.94; their evenness, 0.82. The following species were dominant: *Passer domesticus*, *Fringilla coelebs*, *Hirundo rustica*, *Delichon urbica* and *Carduelis chloris*.

(3) In the non-breeding periods, 52 species were ascertained in the village Diana; their density fluctuated between 133 individuals per 10 hectares in winter and 339 individuals per 10 hectares in late summer. The species diversity oscillated within the limits 2.09–2.57; their evenness was 0.56–0.71. The dominant species during the individual periods included *Passer domesticus*, *Carduelis chloris*, *Parus major*, *P. caeruleus*, *Delichon urbica*, *Carduelis carduelis*, *Emberiza citrinella*, *Pyrrhula pyrrhula* and *Sitta europea*.

In the village Kateřina, 63 species were found in the non-breeding periods; the density of birds fluctuated between 67 individuals per 10 hectares in winter and 398 individuals per 10 hectares in late summer; the species diversity oscillated from 2.31 to 2.80; their evenness, from 0.69 to 0.78. The dominant species in the individual periods included *Passer domesticus, Carduelis chloris, C. carduelis, Hirundo rustica, Delichon urbica, Parus major, P. caeruleus* and *Emberiza citrinella.*

(4) There are differences between both villages as regards the composition of their bird synusia both during the breeding period (a higher share of hollow-breeders, of *Fringilla coelebs, Carduelis chloris* and *Delichon urbica,* a lower share of *Passer domesticus* and *P. montanus*) and during the non-breeding periods at Diana (differences in series of dominant species, in the frequency of bird occurrence, in the density mainly in winter and autumn). The differences are not very conspicuous; for the most part they can be explained by the differences of the habitats closely surrounding the villages. A comparison of data obtained in the villages investigated by us (which are situated in a woodland), with those obtained by Hudec (1973) in villages situated in an open agricultural country, reveals distinct differences in the representation of some species in the breeding synusium *(Passer domesticus, P. montanus, Carduelis chloris, Fringilla coelebs, Parus major).* It seems that the character of the wider environs in which the respective village lies is of significant and probably decisive influence upon the composition of its avifauna.

Резюме

1. В 1974—1976 гг. проводились в двух населенных пунктах Катержинской котловины Чешского леса (югозападная Чехия, Таховский район) наблюдения за птицами. В гнездовом периоде проводились наблюдения гнездовых районов, в мимогнездовом периоде проводились подсчеты птиц на трассах их перелетов. Оба населенных пункта расположены в лесистой местности с мало развитым уровнем сельского хозяйства, один из этих населенных пунктов (Катержина) расположен по соседству с полями, другой (Диана) на окраине леса.

2. Плотность населения сообщества птиц в гнездовом периоде в населенном пункте Диана составляла 15,2 пар на 1 га, здесь гнездились 32 вида птиц, изменчивость составляла 2,79 и „species eveness" — 0,80. Доминирующими видами оказались *Passer domesticus, Fringilla coelebs, Delichon urbica, Carduelis chloris, Turdus merula* и *Parus major.*

В населенном пункте Катержина гнездились 34 вида птиц, их общая плотность составляла 9,6 пар на 1 га, изменчивость — 2,94 и „species eveness" — 0,82. Доминирующими видами оказались: *Passer domesticus, Fringilla coelebs, Hirundo rustica, Delichon urbica* и *Carduelis chloris.*

3. В мимогнездовом периоде было в населенном пункте Диана обнаружено 52 вида птиц, их плотность колебалась от 133 особей но 10 га зимой до 339 особей на 10 га поздным летом. Изменчивость колебалась от 2,09 до 2,57, „species eveness" — от 0,56 до 0,71. К доминирующим видам в отдельные периоды относились *Passer domesticus, Carduelis chloris, Parus major, P. caeruleus, Delichon urbica, Carduelis carduelis, Emberiza citrinella, Pyrrhula pyrrhula* и *Sitta europaea.*

В населенном пункте Катержина было в мимогнездовом периоде определено 63 вида птиц, плотность населения колебалась от 67 особей на 10 га, зимой до 398 особей на 10 га поздним летом, изменчивость колебалась от 2,31 до 2,80 и „species eveness" от 0,69 до 0,78. К доминирующим видам в отдельные периоды относились *Passer domesticus, Carduelis chloris, C. carduelis, Hirundo rustica, Delichon urbica, Parus major, P. caeruleus* и *Emberiza citrinella.*

4. Между двумя исследуемыми населенными пунктами существует определенное различие состава сообществ птиц в гнездовом и в мимогнездовом периодах. Различия оказались не слишком выразительными (самым выразительным оказалась различная доля *Passer domesticus)* и очевидно являются последствием разного типа биотопов непосредственно окружающих оба населенных пункта. Если сравнить наши заключения, полученные в населенных пунктах расположенных преимущественно в лесистой местности с заключениями полученными Гудцем (1973) в населенных пунктах расположенных в открытой местности — в полях, то различия оказываются существеннее. Кажется, что характер ландшафта, в котором населенный пункт расположен, имеет для состава сообщества гораздо большее значение, чем характер непосредственно окружающей среды.

BALOGH, J., 1958: Lebensgemeinschaften der Landtiere. *Berlin–Budapest, 560 pp.*

BENSON, H. B. G. & WILLIAMSON, K., 1972: Breeding birds of a mixed farm in Suffolk. *Bird Study, 19(1): 34–50.*

BERTHOLD, P., 1976: Methoden der Bestandserfassung in der Ornithologie: Übersicht und kritische Betrachtung. *J. Ornithol., 117(1): 1–69.*

BRTEK, V. & FERIANCOVÁ-MASÁROVÁ, Z., 1969: Vtáctvo južnej časti Malých Karpát [The avifauna of the southern part of the Malé Karpaty Mountains]. *Biol. Práce SAV, 15(6): 9–76* (in Slovak).

BULL, A. L., MEAD, C. J., WILLIAMSON, K., 1976: Bird-life on a Norfolk farm in relation to agricultural changes. *Bird Study, 23(3): 163–182.*

DIRCKSEN, R. & HÖHNER, P., 1963: Quantitative ornithologische Bestandsaufnahme im Raum Ravensburg-Lippe. *Abh. Landesmus. Naturk. Münster, 25(3): 1–111.*

ENEMAR, A., 1959: On the determination of the size and composition of a passerine bird population during the breeding season. A methodological study. *Vår Fågelvärld, 2: 1–114.*

FERIANC, O., 1967: Vtáčie synúzie biotopov Blatskej nížiny [The bird synusia of habitats of the Blatská nížina Lowland]. *Acta Fac. Rer. Nat. Univ. Comen., Zool., 12: 193–246* (in Slovak).

FERIANC, O., 1968: Vtáctvo Liptovskej kotliny [The avifauna of the Liptovská kotlina Valley]. *Acta Fac. Rer. Nat. Univ. Comen., Zool., 14: 137–194* (in Slovak).

FERIANCOVÁ-MASÁROVÁ, Z., 1963: Vtáky štyroch biotopov okolia Oravskej priehrady [The birds of four habitats in the environs of the Orava valley reservoir]. *Biológia, 18(1): 45–67* (in Slovak).

FERIANCOVÁ-MASÁROVÁ, Z., 1968: Vtáčie společenstvá západnej časti Liptova (transekt Choč–Chabenec) [The bird communities of the western part of Liptov (the transect Choč–Chabenec)]. *Biol. Práce SAV, 14(5): 105–154* (in Slovak).

HAVLÍN, J., 1975: Zur Erkenntnis der Artendiversität und Dominanz bei verschiedenen Typen menschlicher Siedlungen. *Zool. Listy 24(1): 43–63.*

HUDEC, K., 1973: Die Vogelsynusie im dörflichen Milieu zweiter Gemeinden. *Zool. Listy, 22(4): 347–362.*

HUDEC, K., 1976: Der Vogelbestand in der städtischen Umwelt von Brno (ČSSR) und seine Veränderungen. *Acta Sc. Nat., Brno, 10(11): 1–56.*

LENZ, M., 1971: Zum Problem der Erfassung von Brutvögelbeständen in Stadtbiotopen. *Vogelwelt, 92(2): 41–52.*

MILDENBERGER, H., 1950: Untersuchungen über die Siedlungsdichte der Vögel in der ackerbaulich genutzten Kulturlandschaft. *Bonn. Zool. Beitr., 1(2–4): 221–238.*

NOVIKOV, G. A., 1953: Praktikum polní ekologie suchozemských obratlovců [The handbook of field ecology of terrestrial vertebrates]. *ČSAV Praha, 252 pp.* (in Czech).

ODUM, E. P., 1977: Základy ekologie [Fundamentals of ecology]. *ČSAV Praha* (in Czech).

PEITZMEIER, J., 1950: Untersuchungen über die Siedlungsdichte der Vogelwelt in kleinen Gehölzen in Westfalen. *Natur u. Heimat, Münster, 10: 30–37.*

PIKULA, J., 1968: Dominance among the members of the avian synusy of forest biocenoses. *Zool. Listy, 17(13): 279–293.*

PIKULA, J., 1976: Metodika výzkumu hnízdní bionomie ptactva [The methods of research of the breeding biology of birds]. *SZN Praha, 253 pp.* (in Czech).

TOMIALOJC, L., 1968: Podstawowe metody badań ilosciowych awifauny legowej obszarów zadrzewionych i osedli ludzkich [The fundamental methods of quantitative research of birds breeding in woods and human settlements]. *Not. Ornithol., 9(1–2)* (in Polish).

TURČEK, F. J., 1956: Úvod do kvantitatívného výskumu populácií vtákov i cicavcov [An introduction into a quantitative research of populations of birds and mammals]. *SAV Bratislava* (in Slovak).

TURČEK, F. J., 1958: Ekologické rozšírenie vtákov a cicavcov oblasti Banskej Štiavnice ako prechodovej zóny [The ecological distribution of birds and mammals in the district of Bánská Štiavnica]. *Biol. Práce SAV, 4(8): 5–43* (in Slovak).

TURČEK, F. J., 1971: Synuzie suchozemských stavovcov hornej časti Liptovskej kotliny [The synusia of terrestrial vertebrates of the upper part of Liptovská kotlina Valley]. *Quest. Geobiol., 9: 43–73* in Slovak).

WILLIAMSON, K., 1971: A bird census of a Dorset dairy farm. *Bird Study, 18(2): 80–96.*

Critical revision by RNDr. *J. Pikula*, CSc.

Author's address:

RNDr. Pavel ŘEPA,

Department of Zoology, District Museum, tř. Míru 447, 347 01 Tachov, ČSSR.
