# Supplementary material for: SPARSE 1.0: a template for databases of species inventories, with an open example of Czech birds
Source: Biodivers Data J. 2023 Nov 23;11:e108731. doi: 10.3897/BDJ.11.e108731 (PMC10690794; doi:10.3897/BDJ.11.e108731)
Supplement: Supplementary material 1 — SPARSE 1.0 - all database files as on the date of submission [file bdj-11-e108731-s001.zip › readme.html]

readme


# 1 SPARSE - SPecies AcRoss ScalEs (version 1.0)

**Authors:** Kateřina Tschernosterová, Eva Trávníčková,
Florencia Grattarola, Clara Rosse, Petr Keil

**Contact email:** keil@fzp.czu.cz

## 1.1 License

SPARSE is under CC-BY license (https://creativecommons.org/about/cclicenses/). Users of
the Czech bird data in this database should cite the original
publications (studies) in which the inventories were first published. A
BibTeX file with all the references is in this folder, and can be loaded
to common reference managers such as Zotero or JabRef. Users of the
SPARSE framework, or those who modify it, should cite Keil *et
al.* (2022) paper in **Biodiversity Data Journal**.

---

## 1.2 Structure of the folder

Folders:

- `/code`- scripts that we use to process the data
- `/code/shapefile_linking` - linking shapefiles with
  database queries
- `/code/study_selection_algorithm` - selecting priority
  studies for digitization
- `/code/taxon harmonization` - processing and harmonizing
  taxonomy
- `/CZ_border_shapefile` - shapefile of Czech borders - for
  plotting purposes
- `/SPARSE_shapefiles` - shapefiles of the points, lines,
  and polygons representing surveyed sites

Files:

- `/INPUT_data-template-empty` - empty .xlsx template that
  we used during the first step of data digitization. These .xlsx files
  were then imported to MS Access
- `/INPUT_data-example*` - example study (.pdf) and
  pre-filled template (.xlsx) corresponding to the study
- `/SPARSE.accdb` - the main MS Access file with the four
  core tables
- `/SPARSE_bibliography.bib` - BibTeX bibliography with all
  the digitized studies
- `/SPARSE_definitions.xlsx` - detailed descriptions of all
  fields in the main MC Access file

---

## 1.3 Overview

Hi. This is SPARSE database. It stores data on species composition of
sites in space and through time, where multiple species had been
recorded during the surveys. Thus, SPARSE does not contain presence-only
point observations such as those stored in e.g. GBIF. Examples of data
stored in SPARSE are reserve inventories, point surveys, transects,
commercial surveys prior to construction activities, checklists and
redlists in administrative units such as counties, states, or countries.
SPARSE is currently implemented in MS Access, with additional GIS data
stored in ESRI shapefiles, and with the original raw data provided as
.pdf and .xlsx files.

Full descriptions and details are provided in the
**Biodiversity Data Journal** manuscript.

### 1.3.1 Long-term goals

- To create infrastructure for management of “inventory” data in our
  research group, with emphasis on temporal change and data
  heterogeneity.
- The database should have simple structure, so that others can use it
  or copy it, without a detailed knowledge of databases or spatial
  data.
- The database structure should work for different groups of
  organisms, and in different regions of the world.
- For our group, to learn basics of biodiversity databases, standards,
  and spatial data management.
- To actually have the data for selected groups and regions. The first
  priority is to digitize data on Central European Birds, so that they can
  complement other data that we have for this taxon and region.
- To create a backbone for a bigger database of heterogeneous
  biodiversity data covering Europe, United States, and potentially other
  regions.

### 1.3.2 What makes SPARSE unique?

- It is open, free, and simple.
- It is spatially explicit, and can accommodate complex spatial
  structures and multiple scales, including complex nested sampling
  designs where multiple sites within a larger area have been surveyed,
  and this larger area can again be nested in an even larger region.
- It works well with data on temporal change, i.e. with repeated
  sampling events at a single site.
- It explicitly considers variation in sampling methods and effort, it
  should work for different regions and taxa.
- It uses a combination of Humboldt core and Darwin core
  standards.

---

### 1.3.3 Progress summary

The very first idea was originally conceived in August 2020, at iDiv,
Leipzig, by Petr Keil, and first attempts for implementation were done
together with Clara Rosse, with the backing of Jonathan M. Chase. In the
fall of 2021, a brand new effort had started at the Department of
Spatial Sciences at Czech University of Life Sciences in Prague, where
Petr Keil relocated to start his tenure-track position. During summer
and fall 2021 we have (with Eva Trávníčková, Kateřina Tschernosterová,
and Flo Grattarola) designed a new structure from scratch, matched it
with Darwin Core and Humboldt core standards, and implemented it in MS
Access.

---

## 1.4 Opening SPARSE

**If you have MS Windows and MS Access**

The primary way to open SPARSE is on a MS Windows machine with *MS
Access* installed. We have created SPARSE on MS Access version 2309,
so this is the version on which everything works (double checked by
several users).

**If you have MS Windows but not MS Access**

It should be possible to open SPARSE in free *LibreOffice
BASE*. Fire up the program, choose `connect to database`
and choose `MS Access`.

**If you have Ubuntu Linux**

You can list and extract individual tables from SPARSE.accdb (tested
on Ubuntu 20.04 LTS).

1. install the mdbtools package:
   `sudo apt install mdbtools`
2. list all tables in SPARSE:
   `mdb-tables SPARSE.accdb`
3. export any table to .csv, for example the 1\_DATASET table:
   `mdb-export SPARSE.accdb 1_DATASET > 1_DATASET.csv`

**If you have a Mac with no MS Access**

We haven’t tested this option yet.

---

## 1.5 Acknowledgements

SPARSE has been supported by funding from Research Excellence in
Evnironmental Sciences (REES) provided by Czech University of Life
Sciences. SPARSE has also received funing from the European Union (ERC,
BEAST, 101044740). Views and opinions expressed are however those of the
author(s) only and do not necessarily reflect those of the European
Union or the European Research Council Executive Agency. Neither the
European Union nor the granting authority can be held responsible for
them.
