# Supplementary figures and images for: SPARSE 1.0: a template for databases of species inventories, with an open example of Czech birds
Source: Biodivers Data J. 2023 Nov 23;11:e108731. doi: 10.3897/BDJ.11.e108731 (PMC10690794; doi:10.3897/BDJ.11.e108731)

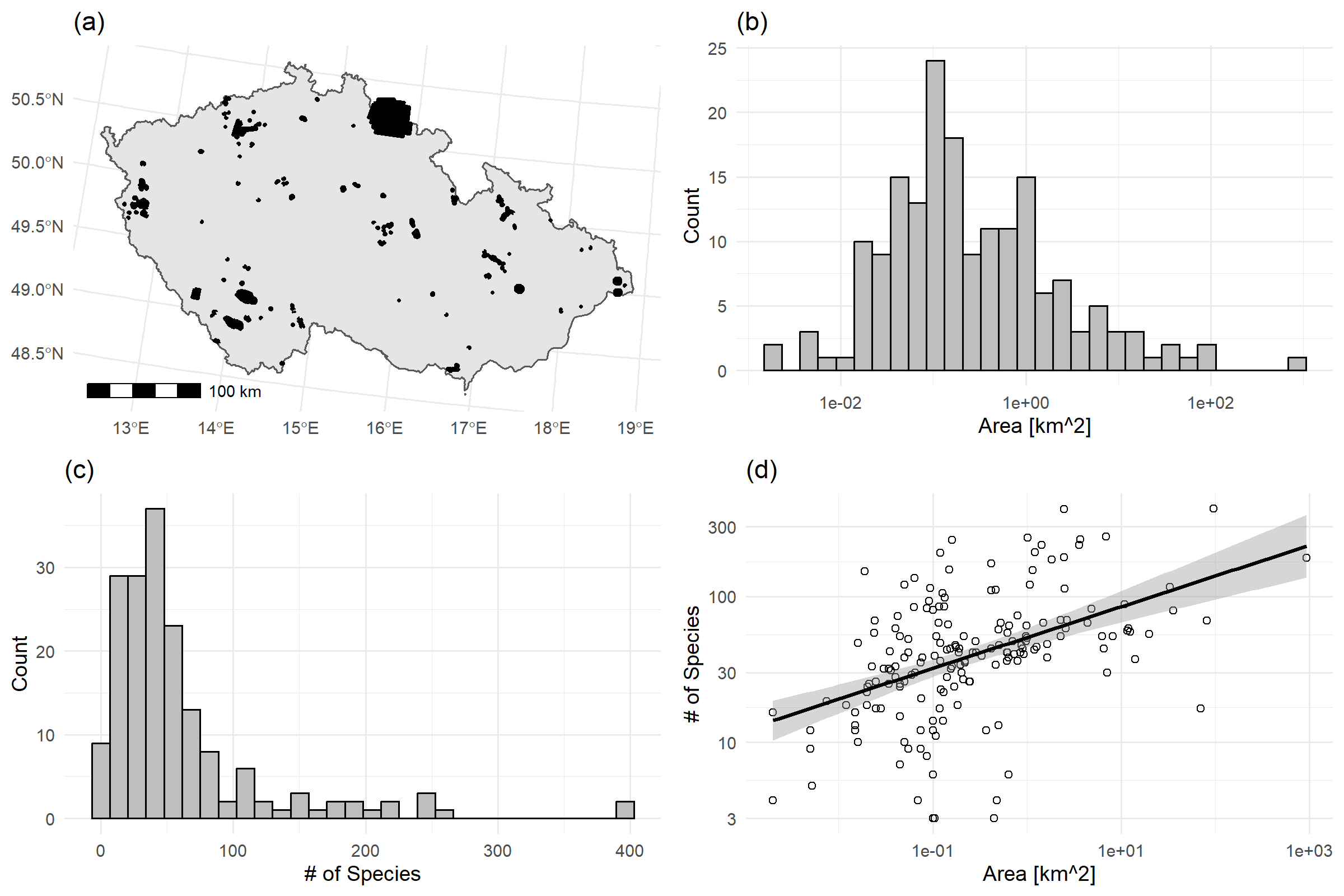

Supplement: Supplementary material 1 — SPARSE 1.0 - all database files as on the date of submission [file bdj-11-e108731-s001.zip › code/shapefile_linking/Fig2.png]

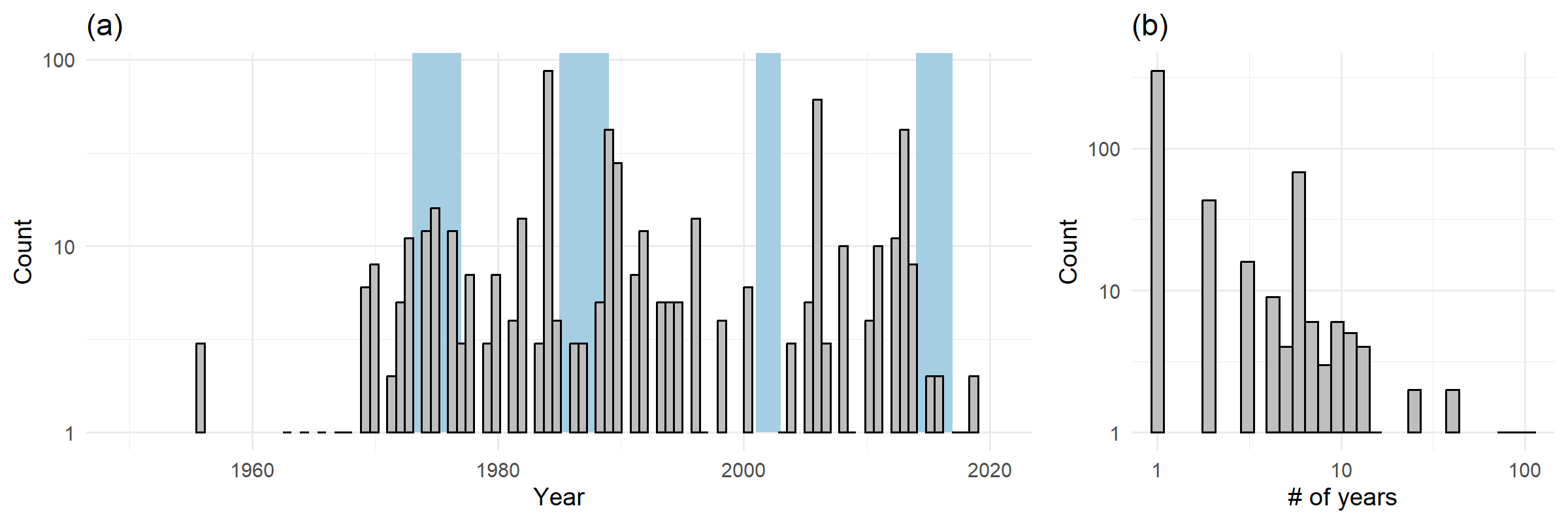

Supplement: Supplementary material 1 — SPARSE 1.0 - all database files as on the date of submission [file bdj-11-e108731-s001.zip › code/shapefile_linking/Fig3.png]
